# HHIP Overexpression Suppresses Human Gastric Cancer Progression and Metastasis by Reducing Its CpG Island Methylation

**DOI:** 10.3389/fonc.2020.01667

**Published:** 2020-12-22

**Authors:** Yu Song, Jianchen Tu, Yanan Cheng, Fang Zhou, Peilin Liu, Shuangshuang Zhou, Yongjun Gu, Yang Sun

**Affiliations:** ^1^Department of Oncology, The First People Hospital of Zhangjiagang City, Soochow University, Zhangjiagang, China; ^2^Department of Gastrointestinal Surgery, The First People Hospital of Zhangjiagang City, Soochow University, Zhangjiagang, China

**Keywords:** HHIP, hedgehog signaling, gastric cancer, methylation, metastasis, growth

## Abstract

Human hedgehog-interacting protein (HHIP), a negative regulator of hedgehog (HH) signaling pathway, has been reported to be dysregulated in many types of cancer, including gastric cancer. However, the inhibitory role of HHIP as well as the underlying molecular mechanism of HHIP regulation in gastric cancer haven’t been fully elucidated yet. In this study, we demonstrated that HHIP overexpression significantly suppressed the proliferation and invasion of AGS cells evaluated by 3-(4,5-dimethylthiazol-2-yl)-2,5-diphenyltetrazolium bromide (MTT) and transwell assays, respectively. Interestingly, methylation-specific polymerase chain reaction (MS-PCR, MSP) showed that HHIP overexpression dramatically decreased its *de novo* promoter methylation levels in AGS cells. Furthermore, HHIP expression was higher in adjacent non-cancerous tissue compared to matched gastric cancer tissue. High HHIP level was negatively correlated with metastasis (*p* = 0.035) but not local recurrence (*p* = 0.58). Taken together, our study suggested that HHIP can modulate gastric cancer progression and metastasis via regulation of its *de novo* promoter methylation levels in a feedback manner. Lower HHIP levels is positively associated with gastric cancer metastasis, which not only indicates HHIP could be served as a protective marker for gastric cancer, but also suggests restoring HHIP expression might be a potential therapeutic strategy for clinical treatment.

## Introduction

The classical hedgehog (HH) signaling pathway includes sonic hedgehog (SHH), Indian hedgehog (IHH), and desert hedgehog (DHH). It has been reported that HH ligand can lead to Smoothened (SMO) activation by binding with Patched (PTCH), followed by nuclear translocation of Gli family and then upregulation of their downstream target genes (1–3). Lots of evidences have shown that abnormal activation of HH signaling pathway contributes to tumor progression, including gastric cancer (4, 5). However, the potential molecular mechanism of HH signaling, especially the dysregulation of Human hedgehog-interacting protein (HHIP) gene, is largely uncertain in gastric cancer.

As a negative regulator of the HH signaling pathway (6–8), HHIP plays crucial roles in tumorigenesis. Decreased HHIP expression has been identified in many types of cancer and is associated with hyperactivation of HH signaling pathway which directly promotes cancer progression (9–11). Consistently, we have already found that HHIP expression is downregulated in gastric cancer (12), however, it is still unknown of the underlying mechanism which causes low expression of HHIP in gastric cancer cells.

To this aim, we have investigated the biological function as well as the potential mechanism of HHIP regulation in gastric cancer in this study. Our results showed that HHIP overexpression significantly suppressed proliferation and migration of gastric cancer cells. Moreover, we also identify an interesting feedback loop functions as a critical event to finely regulate HHIP expression: HHIP overexpression can decrease the CpG island methylation status on its *de novo* promoter, which further inhibits gastric cancer progression and metastasis.

## Materials and Methods

### Patient Tissue Specimens

All the specimens, including 52 paired adjacent non-cancerous tissue and gastric cancer tissue, were collected from Department of Surgery, The First People Hospital of Zhangjiagang City, Soochow University (Jiangsu, China) between 2015 and 2018. All surgically specimens were immediately frozen in liquid nitrogen after removal from the patients. Specimens were inspected by at least 2 professional pathologists and classified according to tumor-node-metastasis (TNM) staging of UICC (International Union against Cancer) in 2020. Patients included 28 males and 24 females with an age range from 36 to 72 years (median 60.82 years). 18 cases were stage I patients, 16 cases were stage II, and 38 cases were stage III patients (Table 1).

**TABLE 1:** Clinical features of patients.

Clinical features	Total
**Sex**	
Male	28
Female	24
Age	
<50	16
≥50	36
**Pathological type**	
Adenocarcinoma	52
TNM stage	
I	18
II	16
III	18

All specimens with patient consents and approval were obtained from the Institute Research Ethics Committee.

### Reagents and Cell Culture

The Lenti-X HTX lentiviral packaging kit and lentiviral vector PlvxDsRed-monomer-nl were purchased from Takara (Japan). Gastric cancer cell line AGS was purchased from the Shanghai Institute of Life Science Cell Information Center of the Chinese Academy of Sciences (Shanghai, China) and cultured with RPMI-1640 (Invitrogen, Carlsbad, CA, United States) including 10% fetal bovine serum (FBS), 100 μg/mL streptomycin and 100 U/mL penicillin.

### Lentiviral Vector Construction and Transduction

The full-length fragment of HHIP was amplified by Polymerase chain reaction (PCR) and then was cloned into lentiviral vector PlvxDsRed-monomer-nl (LV-HHIP). Empty vector PlvxDsRed-monomer-nl was used as the negative control (LV-CON). Lentivirus expressing LV-CON or LV-HHIP was produced as described previously (12). AGS cells were cultured in six-well plates (5 × 10^4^ cells/well) until the confluence reached to 70%, then equal volume of lentivirus expressing LV-CON or LV-HHIP were added into each well for infection. After 72 h, cells were observed under a fluorescence microscope and then harvested for the following experiments.

### Cell Proliferation Assay

Cell proliferation was inspected by the 3-(4,5-dimethylthiazol-2-yl)-2,5- diphenyltetrazolium bromide (MTT) assay. Cells were cultured in 96-well plates (4 × 10^3^/well), followed by lentiviral infection on the next day. After 48 h, 20 μL MTT (5 mg/mL) was added into each well and then incubated for 4 h. Replaced the medium in each well with 150 μL dimethylsulfoxide (DMSO) to dissolve formazan. Finally the optical density values were measured by a microplate spectrophotometer at 492 nm.

### RNA Isolation and Reverse Transcription (RT)-PCR

Total RNA was isolated by TRIzol reagent (Shanghai Jingmei Bio Engineering, China) then converted to cDNA (the SuperScript III First-Strand Synthesis System; Invitrogen). The PCR primers and conditions had described before (12) (Table 2).

**TABLE 2:** Primers used for realtime-PCR, methylation-specific PCR and bisulfite- sequencing PCR.

Method	Primers	Sequence	Length (bp)	Temp (°C)
Realtime-PCR	HHIP	5′-CTGCTTCTGTATTCAGGAGGTT-3′	229	55
		5′-GGGATGGAATGCGAGGCTTA-3′		
	β-actin	5′-AGAGCTACGAGCTGCCTGAC-3′	184	60
		5′-AGCACTGTGTTGGCGTACAG-3′		
BSP	HHIP	5′-GGGGAGGAGAGAGGAGTTTG-3′	243	60
		5′-CCCRACRACCTCCCTACTAC-3′		
	β-actin	5′-AGAGCTACGAGCTGCCTGAC-3′	184	60
		5′-AGCACTGTGTTGGCGTACAG-3′		
MSP	Methylation	5′-GTAGTAGTCGGGTAGTTTCGGAATTTTC-3′	190	60
		5′-AAAAACGACTAACCGCGACG-3′		
	Non-methylation	5′-AGTAGTTGGGTAGTTTTGGAATTTTTGG-3′	188	60
		5′-AAAAACAACTAACCACAACA-3′		

*HHIP, hedgehog-interacting protein; BSP, bisulfite-sequencing PCR; and MSP, methylation-specific PCR.*

### Methylation-Specific PCR and Bisulfite-Sequencing PCR to Detect HHIP Gene Methylation

Primers for methylation-specific PCR (MSP) and bisulfite-sequencing PCR (BSP) analysis to evaluate HHIP promoter methylation status were as described previously (4) and listed in Table 2. The whole procedure was according to the manufacturer’s instruction. Ten clones were randomly selected from each sample for DNA sequencing by Sangon Co. Ltd. (Shanghai, China).

### Transwell Assay

Transwell assays were performed according to the manufacturer’s instructions. AGS cells in serum-free medium were seeded (5 × 10^4^/mL/well) inside the chamber, while 500 μL culture medium containing 10% FBS was added into the outside of the chamber in each well of 24-well plate. After 24 h, non-invasive cells were removed from the upper surface of the membrane, while the invasive cells were fixed in methanol (10 min) and then stained with 0.1% crystal violet hydrate (Sigma, St. Louis, MO, United States; 30 min). After air-drying, the membrane was mounted on the slide and examined using microscope.

### Western Blot Analysis

Total cell lysates were harvested via radioimmunoprecipitation assay buffer (Cell Signaling Biotechnology), then quantified by a bicinchoninic acid protein assay kit (Pierce). Equal proteins were separated on 10% protein gels (Millipore, Bedford, MA, United States). After transferring, the PDVF membranes were blocked with 5% skimmed milk (w/v) at room temperature (2 h) and then incubated with rabbit anti-Flag antibody (1:2000 dilution; Santa Cruz Biotechnology, Santa Cruz, CA, United States) overnight (4°C), followed by incubation with secondary antibodies. The immunoreactive bands were detected with the ECL plus chemiluminescence kit (Beyotime Institute of Biotechnology, Haimen, China).

### Statistical Analysis

SPSS version 16.0 software (SPSS, Inc., Chicago, IL, United States) was used to analyze the results. All measurement data were expressed as the mean ± standard deviation. Comparisons were made by the *t* test between two groups or by one-way analysis of variance among multiple groups. Follow-up data were collected by a specially-assigned person for 3 years. Metastasis free time means the time from the beginning of treatment to the distant metastases. Local recurrence free time means the time from the beginning of treatment to local recurrence. *P* < 0.05 was statistically considered significant.

## Results

### Overexpression of HHIP in Human Gastric Cancer Cells by Lentiviral Infection

**FIGURE 1:**
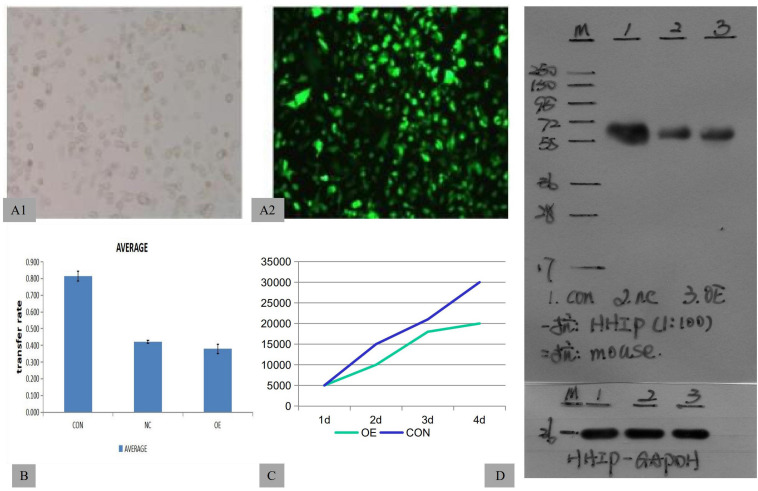
HHIP overexpression was induced by lentivirus-delivered HHIP and inhibited the proliferation of AGS cells. **(A1–2)** The efficiency of lentiviral transduction was monitored by fluorescence microscopy. **(B)** The results of transwell assays showed that the invasive rate of OE group was lowest among three groups (CON>NC>OE). **(C)** MTT assays showed that the cell number of OE group is less than CON group. **(D)** The protein levels of HHIP were assessed by western blotting with an anti-Flag antibody. The size of the band showing the target fusion protein was near 58 kD. M: marker; 1: LV-CON-3FLAG-GFP; 2: LV-NC-3FLAG-GFP; and 3: LV-HHIP-OE-GFP. (NC: negative control, OE; overexpression).

**FIGURE 2:**
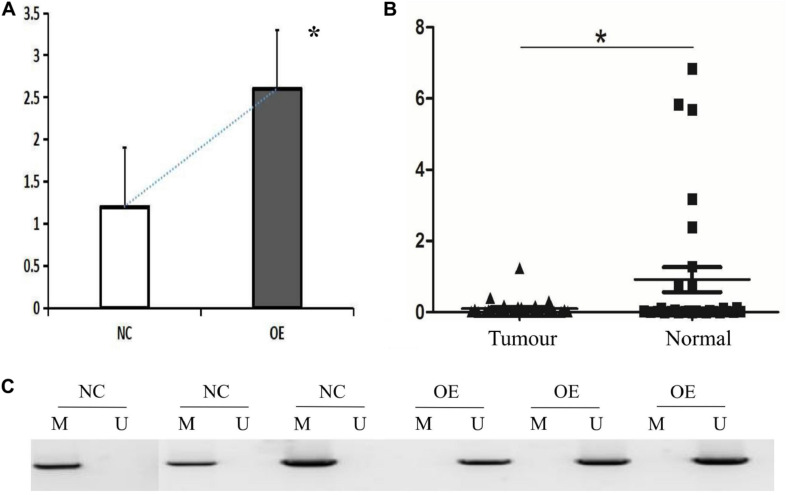
HHIP overexpression reduced methylation levels of *de novo* HHIP promoter in AGS cells and HHIP had lower expression in gastric cancer tissue specimen. **(A)** HHIP overexpression was confirmed by western blot assay in AGS cells; **(B)** HHIP mRNA levels were lower in gastric cancer tissue specimen detected by real-time PCR analysis; **(C)** Comparison of HHIP CpG island methylation proportion in the cells transduced with LV-CON or LV-HHIP. HHIP overexpression reduced methylation levels of HHIP promoter in gastric cancer AGS cells. *p < 0.05.

To assess the roles of HHIP in gastric cancer cell development, we first stably overexpressed HHIP in gastric cancer AGS cells by lentivirus-delivered HHIP (LV-HHIP) or empty control (LV-CON). After infection for 48 h, more than 85% of AGS cells were successfully transduced by LV-HHIP evaluated by immunofluorescence detection (Figures A1–2). Reverse Transcription (RT)-PCR analysis showed that the HHIP mRNA levels were significantly increased in LV-HHIP group (transduced with LV-HHIP), comparing to the control group LV-CON (Figure 2A). Overexpression of HHIP was further confirmed by western-blot analysis at transitionally level (Figure 1D). Overall, these results showed that lentiviral LV-HHIP had efficiently infected AGS cells and indeed increased the expression of HHIP in gastric cancer cells.

### Overexpression of HHIP Inhibits the Proliferation and Invasion of Human Gastric Cancer Cells

To test the role of HHIP overexpression in proliferation and invasion of gastric cancer cells, AGS cells were transduced with LV-HHIP, or LV-CON and cultured for consecutive days (from day 1 to day 6). Cell proliferation rate was monitored by MTT assay on each day following transduction correspondingly. The results showed that compared to LV-CON, HHIP overexpression substantially inhibited proliferation of AGS cells (Figure 1C). Additionally, in comparison to LV-CON cells, LV-HHIP cells significantly demonstrated lower mobility to invade through matrigel-covered-membrane in transwell assays as shown in Figure 1B. Therefore, our data showed that HHIP overexpression suppressed the progression of gastric cancer by inhibiting cell proliferation, migration, and invasion.

### Overexpression of HHIP Inhibits HHIP Promoter Methylation in Human Gastric Cancer Cells

To clarify the potential mechanism caused low HHIP expression in gastric cancer cells, we next try to assess the *de novo* HHIP promoter methylation status. After infection with LV-HHIP or LV-CON for 72 h, the HHIP promoter methylation levels in AGS cells were detected by both MSP and BSP analysis. The MSP results suggested that the PCR products from non-methylated primers increased after HHIP overexpression, while the PCR products from the methylated primers was significantly decreased (Figure 2C). Moreover, the HHIP CpG island methylation proportion was accounted for 99.67% in LV-CON cells, while it was 13.85% in LV-HHIP cells (Table 3). Consistently, the BSP results showed that compare to LV-CON, the methylation level of HHIP promoter was significantly reduced in LV-HHIP cells. Thus, all these data suggested that overexpression of HHIP could inhibits HHIP promoter methylation in human gastric cancer cells in a feedback manner.

**TABLE 3:** The methylation-specific PCR results.

Group	*N*	methylation rate(%)	*p*
LV-CON	3	99.67	<0.05
LV-HHIP	3	13.85	

### HHIP Was Higher in Normal Gastric Tissues and Positively Correlated With Metastasis Free Rate in Gastric Cancer

**FIGURE 3:**
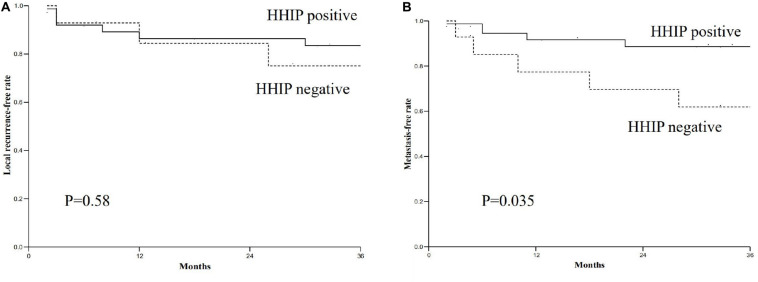
The relationship between HHIP expression levels and local recurrence free rate and metastasis free rate. **(A)** The local recurrence free rate of HHIP positive group was higher than negative group (83.5% via 75%; *p* = 0.58); **(B)** The metastasis free rate of HHIP positive group was higher than negative group (88.6% via 61.9%; *p* = 0.035).

We tested HHIP expression levels in all the specimens from 52 paired gastric cancer tissue and matched adjacent normal tissues by RT-PCR analysis. The results showed that HHIP expression was higher in adjacent normal gastric tissues than in gastric cancer tissues (Figure 2B). After 3 years follow-up, we found that the metastasis free rate was positively correlated with HHIP levels. The metastasis free rate was 88.6% in HHIP positive group, whereas it was only 61.9% in HHIP negative group (*p* = 0.035; Figure 3B). Instead, the local recurrence free rate was not correlated with HHIP levels. The local recurrence free rate was 83.5% in HHIP positive group while 75% in HHIP negative group (via 75%; *p* = 0.58; Figure 3A). Our results suggested that higher HHIP levels showed higher metastasis free rate, which not only hints the protective role of HHIP in gastric cancer development, but also provides evidence to serve HHIP as a potential biological target for gastric cancer treatment.

## Discussion

As we known, HHIP can encode a transmembrane glycoprotein which binds to HH proteins, thereby inhibiting the HH pathway signaling (13, 14). HHIP is located on chromosome 3q31.21-31.3 and HHIP loss could lead to gastrointestinal tumors (15). It has been reported that HHIP was downregulated in gastric cancer (16), which suggests HHIP might function as a tumor suppressor in the gastric tumorigenesis. Consistently, our previous study has just demonstrated that HHIP had lower expression in gastric cancer cells compare to the normal control cells (12). Though the correlation between HHIP and gastric cancer malignance has been studied, the precise function of HHIP as well as the mechanism of HHIP regulation in gastric tumorigenesis remains to be uncertain. In this study, we observed that overexpression of HHIP significantly inhibited the proliferation and invasion of AGS cells, which might provide evidences to further support the tumor suppressor function of HHIP in gastric cancers.

Hedgehog signaling plays an indispensable role in regulation of gastric cancer cell proliferation, migration and invasion (17–19), however, the potential molecular mechanism underlying dysregulation of HH signaling in gastric cancer is still uncertain. Since HHIP is a negative regulator of HH signaling (13), the upregulation of HHIP may play a critical role in suppressing HH signaling pathway in gastric cancer. In this study, we demonstrated that HHIP overexpression significantly decreased *de novo* HHIP promoter methylation levels in AGS cells. Interestingly, our results suggest that there might be a positive feed-back regulating HHIP expression by modulating its own promoter methylation levels in gastric cancer cells. Importantly, we also found that HHIP positive specimens showed higher metastasis free rate of gastric cancer. We speculate that HHIP overexpression inhibits human gastric cancer growth and metastasis via decrease of CpG island methylation levels on its own promoter. Thus, HHIP might be used as a biological target for gastric cancer, which definitely needs more clinical research in future.

Gene therapy mainly depends on the effectively delivery of exogenous DNA into the target cells (20, 21). A lentiviral carrier is a gene therapy vector based on the human immunodeficiency virus and has been developed to infect cells with long-term maintenance *in vivo*. In this study, we applied a “suicide” virus, which infects target cells but does not produce new viral particles in the host cells (22, 23). The constructed HHIP-overexpressing lentivirus demonstrated a high infection rate and produced high levels of HHIP in gastric cancer cells. Importantly, AGS cells infected with HHIP-overexpressing lentivirus led to a significant reduction of cell proliferation and invasion. Our results show that the constructed HHIP-overexpressing lentivirus is a promising therapeutic strategy to treat gastric cancer through downregulation of HH signaling pathway. Further animal experiments are necessary to evaluate the effects of HHIP-overexpressing lentivirus used as a therapeutic agent for gastric cancer treatment.

In summary, we have established a lentiviral vector to overexpress the HHIP gene in gastric cancer cells and found that overexpression of HHIP remarkably inhibited the proliferation and invasion of AGS cells, accompanied by decreased *de novo* HHIP promoter methylation levels in AGS cells. Our study revealed a positive feed-back loop in regulation of HHIP expression in gastric cancer cells. HHIP overexpression might be used to inhibit human gastric cancer growth and metastasis via reducing CpG island methylation on its own promoter. All suggests that HHIP might be an efficient target and overexpression of HHIP by a lentiviral vector might be a promising therapeutic strategy for gastric cancer treatment.

## Data Availability Statement

The raw data supporting the conclusions of this article will be made available by the authors, without undue reservation.

## Author Contributions

All authors contributed to the article and approved the submitted version.

## Conflict of Interest

The authors declare that the research was conducted in the absence of any commercial or financial relationships that could be construed as a potential conflict of interest.
